# Chromosome genome assembly and annotation of the spiny red gurnard (*Chelidonichthys spinosus*)

**DOI:** 10.1038/s41597-023-02357-y

**Published:** 2023-07-12

**Authors:** Yibang Wang, Hui Zhang, Weiwei Xian, Wataru Iwasaki

**Affiliations:** 1 CAS Key Laboratory of Marine Ecology and Environmental Sciences, Institute of Oceanology, Chinese Academy of Sciences, Qingdao, China; 2 University of Chinese Academy of Sciences, Beijing, China; 3 Department of Integrated Biosciences, Graduate School of Frontier Sciences, the University of Tokyo, Kashiwa, Japan

**Keywords:** Rare variants, Ecology

## Abstract

*Chelidonichthys spinosus*, a secondary economic fish, is increasingly being exploited and valued in China. However, overfishing has led to it being recognized as one of the most depleted marine species in China. In this study, we generated a chromosome-level genome of *C. spinosus* using PacBio, Illumina, and Hi-C sequencing data. Ultimately, we assembled a 624.7 Mb genome of *C. spinosus*, with a contig N50 of 13.77 Mb and scaffold N50 of 28.11 Mb. We further anchored and oriented the assembled sequences onto 24 pseudo-chromosomes using Hi-C techniques. In total, 25,358 protein-coding genes were predicted, of which 24,072 (94.93%) genes were functionally annotated. The dot plot reveals a prominent co-linearity between *C. spinosus* and *Cyclopterus lumpus*, indicating a remarkably close phylogenetic relationship between these two species. The assembled genome sequences provide valuable information for elucidating the genetic adaptation and potential molecular basis of *C. spinosus*. They also have the potential to provide insight into the evolutionary investigation of teleost fish and vertebrates.

## Background & Summary

*Chelidonichthys spinosus* (McClelland, 1844), commonly known as spiny red gurnard in the order Scorpaeniformes, is mainly distributed in the Yellow Sea, East China Sea, and South China Sea, and has also been reported in the coastal waters of Japan, North, and South Korea^1^. With the decline of traditional economic fish resources, the fishery community structure has changed significantly. *C. spinosus*, as a secondary economic fish, has been gradually exploited and valued^2^. According to the Food and Agriculture Organization (FAO) estimate, 33.1% of the world’s marine fish stocks were fished at biologically unsustainable levels in 2015^3^. Among them, *C. spinosus* has been recognized as one of the most depleted marine species in China due to overfishing^4^. However, in the northern sea areas of China (such as Haizhou Bay in the middle-southern part of the Yellow Sea), the resource amount of *C. spinosus* has increased, and it has become one of the dominant species in the demersal fish community^5^. This phenomenon may be due to the narrowing and northward movement of *C. spinosus*’s distribution range as a result of climate change^6^.

In addition to its ecological and economic significance, *C. spinosus* possesses unique morphological characteristics. Its snow-white belly, reddish-brown waist, and pair of colorful, flashing green fluorescent large “butterfly wings” under its gills are particularly striking. Underneath the “butterfly wings”, there are three pairs of ballerina-like “feet”. These “feet” are six separate fins, which are highly flexible and contain “taste buds” that aid in locating food^7^. *C. spinosus*’s ability to contract its swim bladder and produce a sound similar to “bubbles” during feeding has earned it the nickname “underwater poet”. These morphological features are believed to be closely linked to its living habits, as *C. spinosus* typically resides in sandy coastal areas and uses its separate fins located below the pelvic fins to crawl along the ocean floor and search for food^8^. The butterfly-like pelvic fins may play a role in predatory or reproductive behavior, though there is still a lack of sufficient evidence to support these assumptions.

As genomics continues to advance, an increasing number of genomes are being sequenced and published. At present, the genomes of approximately 990 fish species have been sequenced and deposited in the NCBI database^9^. However, there is a dearth of genetic information available for the functional genomics and adaptive evolution of *C. spinosus*, as its genome has not been sequenced. Genomic information, as an essential conservation and management tool^10–14^, is necessary for the protection and long-term survival of marine species. Genomic data and resources can greatly enhance our understanding of the species’ diversity and adaptive evolution and can provide a strong foundation for the implementation of effective conservation measures.

**Fig. 1:**
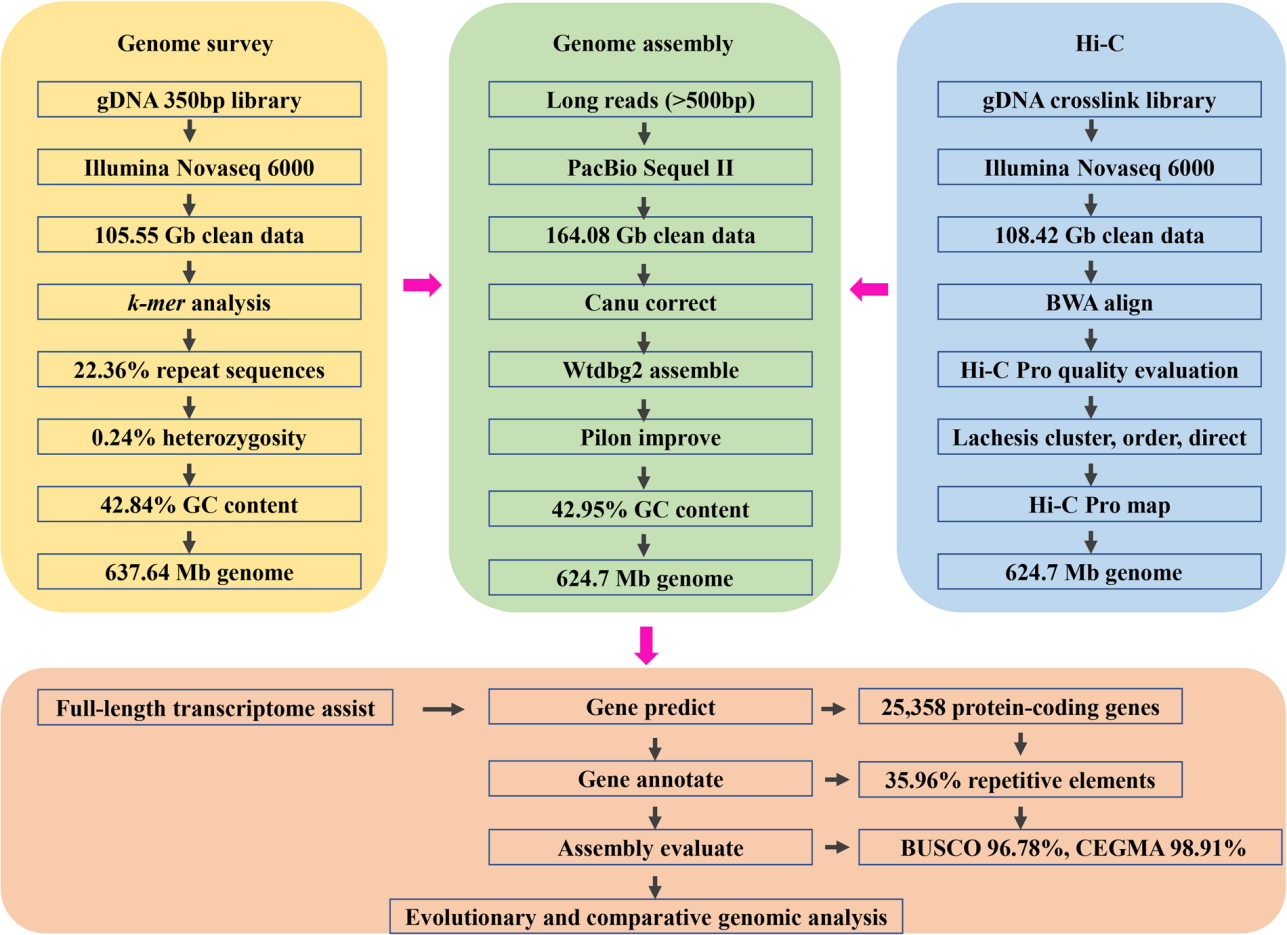
The pipelines overview of *C. spinosus* chromosome-level genome assembly.

In this study, a high-quality chromosome-level genome assembly of *C. spinosus* was generated by Illumina short reads, PacBio long reads, and Hi-C techniques (Fig. 1). The final assembled genome size of *C. spinosus* was 624.7 Mb with an N50 contig length of 13.77 Mb and scaffold N50 length of 28.11 Mb. Furthermore, 99.29% of the initially assembled sequences were anchored on 24 chromosomes. The genome contained 35.96% repeat sequences and 8,235 noncoding RNAs. A total of 25,358 protein-coding genes were predicted, of which 24,072 genes (94.93%) were functionally annotated. The assembled genome sequences can provide valuable information for elucidating the genetic adaptation and potential molecular basis of *C. spinosus*, which can be used to establish more effective management and conservation strategies for this species. Additionally, these genomic data can be also used for future comparative genomics and phylogenetic studies, which may shed light on the genomic evolution and phylogeny of Scorpaeniformes and other teleost fish and vertebrates.

## Methods

### Sample collection and sequencing

**Fig. 2:**
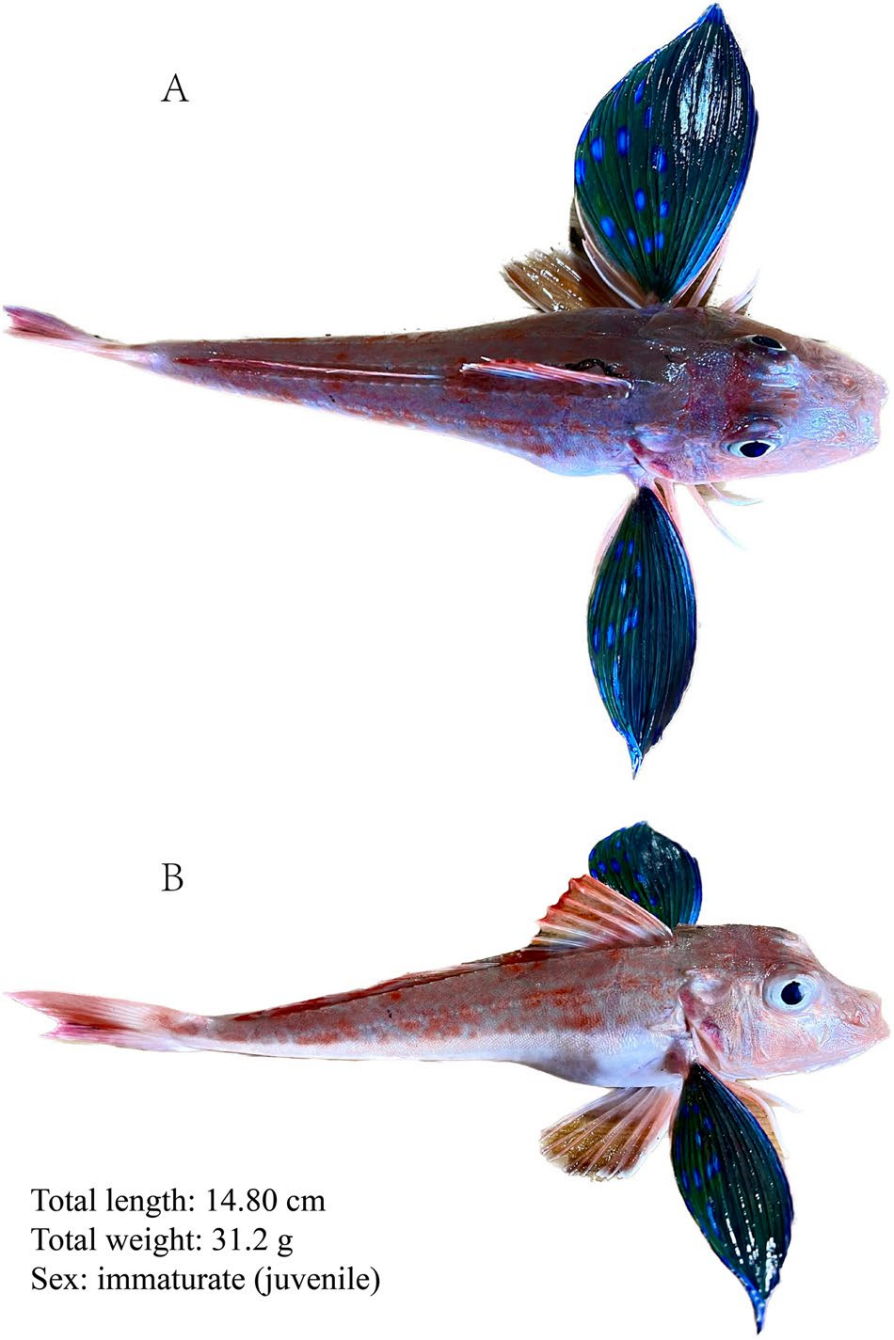
Pictures of *C. spinosus* juvenile used in the genome sequencing and assembly. A represents the top view, and B represents the side view.

Biopsy material for DNA and RNA isolation was obtained from a juvenile spiny red gurnard (*Chelidonichthys spinosus*) collected from Yangtze Estuary, China (Fig. 2). Owing to the immature status of the gonad, the sex could not be determined. All experiments were performed by relevant guidelines and regulations established by the Institutional Animal Care and Use Committee of the Institute of Oceanology, Chinese Academy of Science.

The same specimen was used for genome sequencing (muscle), construction of the Hi-C library (muscle), and transcriptome sequencing of spleen, eyes, brain, kidney, liver, stomach, pelvic fins, and heart. Approximately 500 mg of tissue was dissected and stored in liquid nitrogen before being delivered in dry ice. DNA extraction was performed using an SDS-based analysis method, followed by purification using chloroform. High-quality DNA was used for library preparation and high-throughput sequencing.

For short-read sequencing, one hundred nanograms (ng) of genomic DNA (gDNA) were used to prepare the library. Briefly, gDNA was sonicated to a fragment size of 350 bp by an ultrasonicator and then the library was prepared by using an NEB Ultra DNA library prep kit (NEB, UK) following the manufacturer’s instructions. The library was sequenced on Illumina Novaseq6000 platform (Illumina, Inc., San Diego, CA, USA) using the run configuration 3 × 350 bp for paired-end (PE150) sequencing. A total of 105.55 Gb (165.53 × coverage) of short sequencing reads (clean data) were generated and retained for genome survey on the Illumina sequencing platform (Table 1).

**Table 1:** Sequencing data used for the genome *C. spinosus* assembly.

Sequencing technology	Illumina	PacBio	Hi-C
Clean data (Gb)	105.55	164.08	108.42
Depth (×)	165	262.51	—
GC content (%)	42.84	42.95	42.44
Q20 (%)	96.88	—	96.29
Q30 (%)	92.2	—	91.02

Long-read sequencing was performed using a PacBio II sequencer (Pacific Biosciences, Menlo Park, CA, USA) following the manufacturer’s protocols. Briefly, g DNA was sheared into fragments using the Covaris g-TUBE device, followed by damage-repair, and end-repair using the SMRTbell Damage Repair Kit (PacBio)^15^ on interrupted DNA fragments. The dumbbell connector was then attached, and the fragments were digested by exonuclease. The sequencing library was obtained by screening the target fragments using BluePippin (Sage Science, MA, USA). For the PacBio platform, a total of 164.08 Gb (262.51 × coverage) PacBio long sequencing reads (clean data) with N50 read length of 1,786 bp were obtained after removing adaptors in polymerase reads (Table 1).

The chromosome-level assembly of the *C. spinosus* genome was accomplished by preparing a Hi-C library as per protocol^16^ and sequencing it using the Illumina Novaseq6000 platform. This procedure began by digesting purified DNA from a fresh muscle sample with the HindIII restriction enzyme. Subsequently, this DNA was labeled with Biotin-14-dATP (Thermo Fisher Scientific, USA) through an incubation process, followed by ligation using T4 DNA Ligase. Post an overnight incubation to facilitate reverse cross-linking, the ligated DNA was sheared into fragments ranging between 300 and 700 base pairs. DNA fragments harboring interaction relationships were selectively captured using streptavidin magnetic beads, allowing for the construction of the library. Following library construction, the Qubit 2.0 and Agilent 2100 systems were employed to assess the library’s concentration and insert size. To ensure the library’s quality, its effective concentration was precisely measured using the Q-PCR method. In conclusion, the prepared Hi-C libraries were quantified and sequenced on the Illumina NovaSeq6000 platform (Illumina, USA) using a PE-150 module. This process yielded a total of 108.42 Gb of clean data, determined using the same filter criteria as for short reads (Table 1).

For substantiating transcripts to annotate the genome structure, we carried out RNA-seq on muscle, spleen, eye, brain, kidney, liver, stomach, pelvic fin, and heart samples. This procedure was undertaken by the standard protocol supplied by Oxford Nanopore Technologies (ONT). RNA was extracted from all samples using the Illustra RNAspin Mini RNA Isolation Kit (GE Healthcare, UK), and DNA contamination was eliminated through DNase I treatment. After assessing the quality of a NanoPhotometer® spectrophotometer (Implen, USA), we constructed the RNA-seq libraries in line with the provided protocol. The libraries were then sequenced on the ONT platform (ONT, UK), resulting in a total of 20.42 Gb of clean transcriptome data, as shown in Table 2.

**Table 2:** The transcriptome result of *C. spinosus*.

Sequencing technology	Oxford Nanopore Technologies
Raw data (Gb)	24.89
Clean data (Gb)	20.42
Reads Mean (bp)	1, 266
Reads N50 (bp)	1, 564
Reads Max (bp)	312, 508

### Genome assessment and assembly of *C. spinosus*

**Fig. 3:**
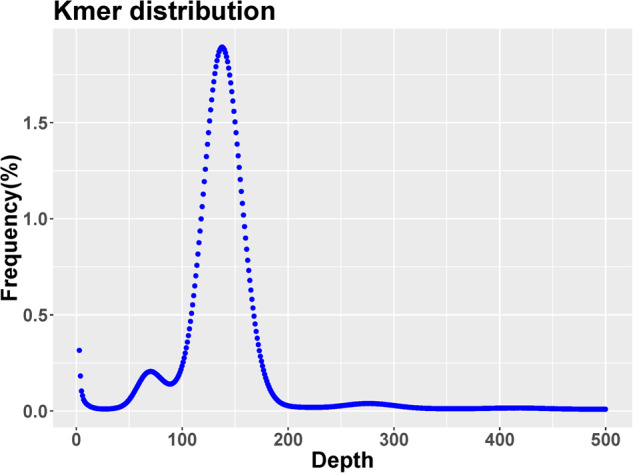
21-mer frequency distribution in *C. spinosus* genome. The X-axis is the *k-mer* depth, and Y-axis represents the frequency of the *k-mer* for a given depth.

The *k-mer* method was used to survey the genomic features of *C. spinosus*. The *k-mer* count histogram (*k* = *21*) was obtained from 105.55 Gb Illumina paired-end sequencing data using Jellyfish v2.9927^17^ and the genome size was estimated using the formula with amendment: G = N _*k*-mer_/Daverage _*k*-mer_, where G is genome size, N _*k*-mer_ is the total number of *k*-mers, Daverage _*k*-mer_ is the average depth of *k-mers*. After removing the *k-mers* with abnormal depth, a total of 91,430,990,545 *k-mers* were obtained with a *k-mers* peak at a depth of 134 (Fig. 3). The genome size of *C. spinosus* was estimated to be 637.64 Mb, and the estimated heterozygosity rate was approximately 0.24% (Table 3).

**Table 3:** The result of *k-mer* analysis.

K_*mer*_	Depth	N_*k-mer*_	Genome size (Mb)	Heterozygous rate (%)	Repeat rate (%)
21	134	91,430,990,545	637.64	0.24	22.36

The 164.08 Gb PacBio long-read data were used for genome assembly using wtdbg2^18^ followed by Quiver^19^ and Pilon^20^ polishing using the 105.55 Gb of Illumina HiSeq clean reads, which produced a 624.7 Mb genome assembly, consisting of 406 contigs with a contig N50 size of 13.77 Mb. In addition, the number and length of the gap are 113 and 11,300 bp (Table 4).

**Table 4:** Assembly statistics of *C. spinosus*.

Type	Contig length (bp)	Scaffold length (bp)	Contig number	Scaffold number	Gap Num	Gap Len (bp)
Total	624,700,534	624,711,834	406	293	113	11300
Max	27,563,186	35,339,233	—	—	—	—
Number > = 1000	—	—	406	293	—	—
N50	13,767,766	28,108,104	—	—	—	—
N90	1,988,450	19,683,350	—	—	—	—

**Fig. 4:**
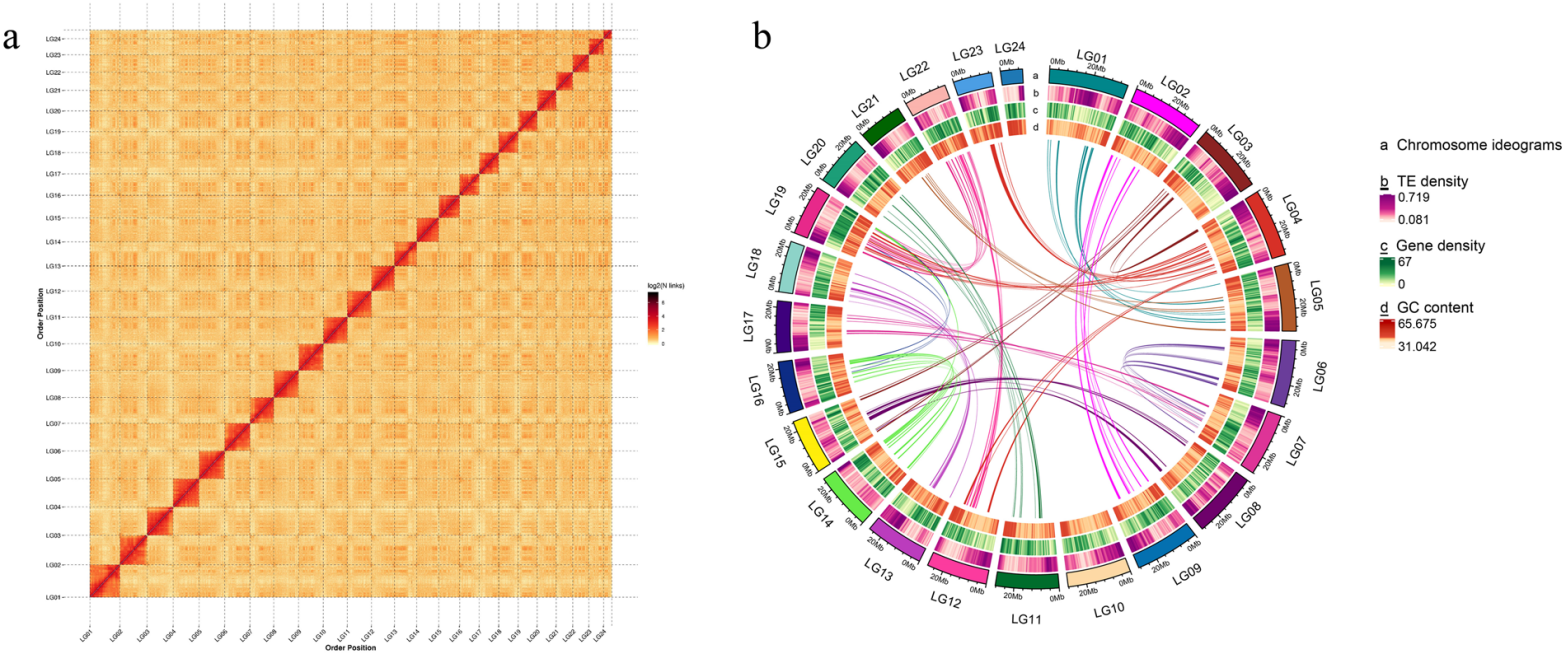
Characteristics of the *C. spinosus* genome. (**a**) Hi-C intra-chromosomal contact map of the *C. spinosus* genome assembly. (**b**) Circos plot of the *C. spinosus* genome assembly.

**Fig. 5:**
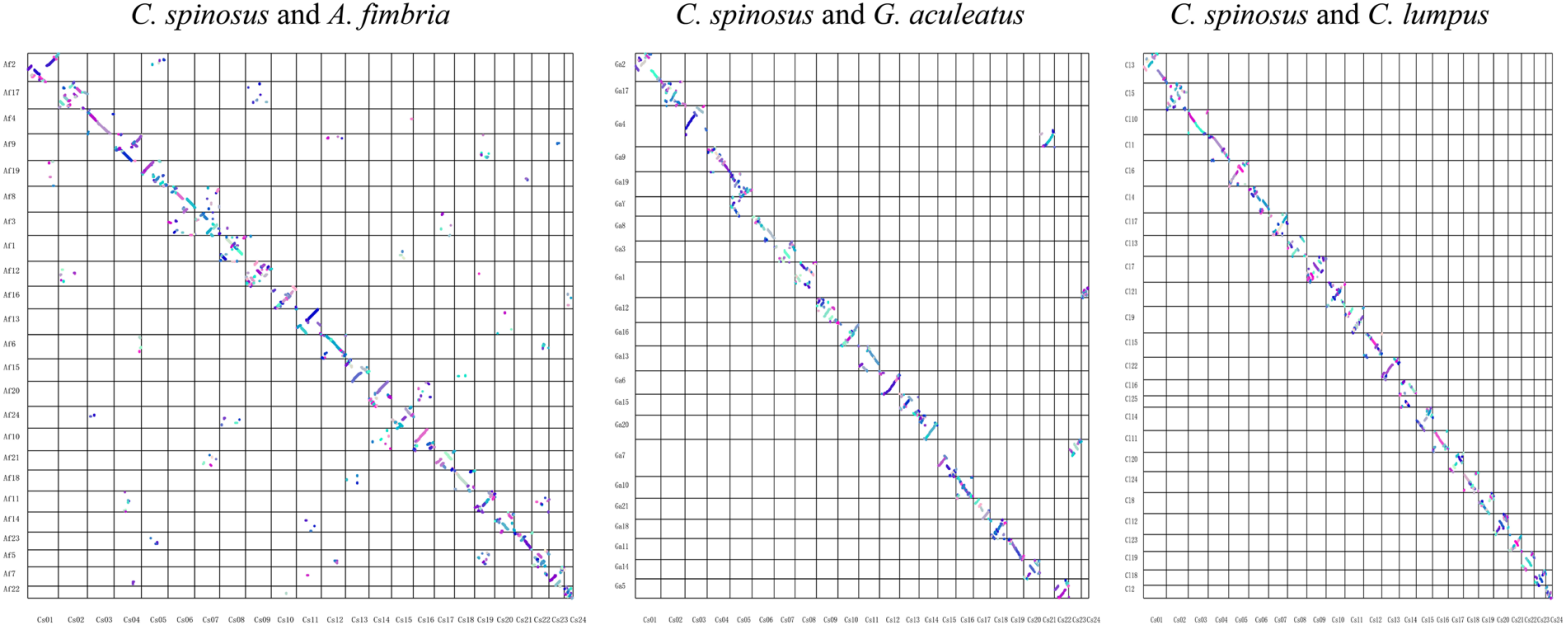
A dot plot depicting the collinearity relationships among *C. spinosus* and its three closest species (*Anoplopoma fimbria*, *Gasterosteus aculeatus*, *Cyclopterus lumpus*).

We employed Hi-C technology to facilitate the chromosome-level genome assembly of the *C. spinosus*. A robust 108.42 Gb of clean data (Table 1) was aligned to the preliminary genome assembly utilizing BWA v0.7.10^21^. Subsequent steps involved the removal of duplications, sorting, and quality control, all carried out via HiC-Pro v2.8.0^22^. For in-depth analysis, we used exclusively those read pairs that were uniquely mapped and valid. Using LACHESIS^23^, the contigs were clustered, ordered, and oriented, resulting in a chromosomal-scale assembly. The parameters deployed in LACHESIS included CLUSTER_MIN_RE_SITES at 59, CLUSTER_MAX_LINK_DENSITY at 2, ORDER_MIN_N_RES_IN_TRUNK at 53, and ORDER_MIN_N_RES_IN_SHREDS at 52. The resulting assembled sequences were further anchored and oriented onto 24 pseudo-chromosomes, employing Hi-C data. These pseudo-chromosomes, ranging in size from 10.45 to 36.62 Mb (Fig. 4 and Table 5), encompassed approximately 99.29% of the total genome. The final *C. spinosus* genome assembly was presented with 293 scaffolds, spanning a cumulative length of 624,711,834 base pairs, a contig N50 of 13.77 Mb, and a scaffold N50 of 28.11 Mb (Table 4). Furthermore, we have produced dot plots depicting the high-quality chromosome assembly genomes of *C. spinosus* and its three closest related species (*Anoplopoma fimbria*, *Gasterosteus aculeatus*, *Cyclopterus lumpus*). These dot plots serve to visually demonstrate the co-linearity relationship among them. Specifically, the dot plot highlights a pronounced co-linearity between *C. spinosus* and *Cyclopterus lumpus*, suggesting a remarkably close phylogenetic relationship between these two species. (Fig. 5).

**Table 5:** The sequence distribution of each chromosome using Hi-C technology.

Group	Cluster Num	Cluster Len	Order Num	Order Len
LG01	6	35617024	4	35338933
LG02	8	32111989	7	32091611
LG03	14	31335771	6	30816615
LG04	13	30931560	4	30412170
LG05	6	30327321	4	30161046
LG06	3	29865028	3	29865028
LG07	25	29598750	7	28107504
LG08	7	29416590	4	29330358
LG09	10	28946362	7	28813697
LG10	6	28274365	5	28234500
LG11	2	28267941	2	28267941
LG12	7	27213928	6	27156097
LG13	18	26801967	9	26195131
LG14	1	25856699	1	25856699
LG15	14	24933336	7	24585908
LG16	12	23354242	9	23231394
LG17	4	22934131	2	22839576
LG18	11	22896109	9	22829349
LG19	2	22474802	2	22474802
LG20	9	22408093	8	22382064
LG21	12	19835980	8	19682650
LG22	3	19028656	3	19028656
LG23	15	17399192	11	17181657
LG24	13	10452535	9	9822798
Total (Ratio %)	221 (54.43)	620282371 (99.29)	137 (61.99)	614706184 (99.10)

Note: Chr01-24 represent 24 chromosomes; Cluster Num: the number of sequences located on a chromosome; Cluster Len: the length of sequence located on a chromosome; Order Num: the number of sequences of the direction can be determined; Order Len: the sequence length of the direction can be determined.

### Repetitive sequence annotation

A combined strategy using homology alignments and de novo searches to identify whole-genome repeats was applied in our repeat annotation pipeline. Transposon element (TE) and tandem repeat were annotated by the following workflows. Firstly, RepeatModeler2 (v2.0.1)^24^ was adopted for ab initio prediction, mainly using two ab initio prediction software RECON (v1.0.8)^25^ and RepeatScout (v1.0.6)^26^. Then full-length long terminal repeat retrotransposons (fl-LTR-RTs) were identified using both LTRharvest (v1.5.9)^27^ (-minlenltr 100 -maxlenltr 40000 -mintsd 4 -maxtsd 6 -motif TGCA -motifmis 1 -similar 85 -vic 10 -seed 20 -seqids yes) and LTR_finder (v1.1)^28^ (-D 40000 -d 100 -L 9000 -l 50 -p 20 -C -M 0.9). The high-quality intact fl-LTR-RTs and non-redundant LTR library were then produced by LTR_retriever (v2.8)^29^. Non-redundant species-specific TE library was constructed by combining the denovo TE sequences library above with the known Repbase (v 19.06)^30^, REXdb (V3.0)^31^^,^ and Dfam (v3.2)^32^ database and RepeatClassifier^24^ was used to classify the prediction results. Final TE sequences in the *C. spinosus* genome were identified and classified by homology search against the library using RepeatMasker (v4.10)^33^. Tandem repeats were annotated by Tandem Repeats Finder and MIcroSAtellite identification tool (MISA v2.1)^34^. The results showed that 35.96% of the *C. spinosus* genome was annotated as repetitive elements, of which transposable elements (TE) were a total length of 183.01 Mb, accounting for 29.28% of the whole genome. Tandem repeats were a total length of 41.75 Mb and represented 6.68% of the whole genome (Table 6).

**Table 6:** The repeat sequence statistics of the assembled genome.

Type	Number	Length	Rate(%)
ClassI: Retroelement	388,036	83,583,913	13.37
LTR/Cassandra	5	325	0
LTR/Caulimovirus	210	22,896	0
LTR/Copia	13,687	1,943,034	0.31
LTR/Gypsy	92,992	24,777,528	3.96
LTR/Pao	11,702	1,881,648	0.3
LTR/Unknown	60,700	16,679,428	2.67
LTR/Viper	98	24,249	0
DIRS	8,673	1,733,410	0.28
LINE	124,198	28,351,818	4.54
LTR/ERV	54,147	5,544,778	0.89
LTR/Unknown	51	3,280	0
SINE	21,573	2,621,519	0.42
ClassII: DNA transposon	639,904	99,422,981	15.91
Academy	3,263	538,417	0.09
CACTA	59,925	7,771,452	1.24
Crypton	11,494	1,248,746	0.2
Dada	1,374	208,963	0.03
Ginger	4,145	499,854	0.08
Helitron	15,297	3,690,150	0.59
IS3EU	3,176	616,530	0.1
Kolobok	23,074	3,356,736	0.54
MITE	34	2,141	0
Maverick	4,168	422,460	0.07
Merlin	1,721	225,323	0.04
Mutator	5,365	546,073	0.09
Novosib	12,076	1,645,215	0.26
P	21,618	4,153,579	0.66
PIF-Harbinger	51,800	10,415,730	1.67
PiggyBac	11,204	1,524,025	0.24
Sola	6,514	804,080	0.13
Tc1-Mariner	51,280	9,590,347	1.53
Unknown	74,567	10,300,517	1.65
Zator	1,431	178,432	0.03
Zisupton	17,691	2,096,228	0.34
hAT	258,687	39,587,983	6.33
srpRNA	1	299	0
ClassIII: Tandem repeats	494,624	41,754,490	6.68
Microsatellite (1–9 bp units)	471,782	13,864,389	2.22
Minisatellite (10–99 bp units)	16,708	18,572,430	2.97
Satellite (> = 100 bp units)	6,134	9,317,671	1.49
Total	1,522,565	224,761,683	35.96

Note: Type: the type of repetitive sequence (Class I: retrotransposons; Class II: DNA transposon; Class III: Tandem repeats); Number: the number of repetitive sequences; Length: the total length of predicted repetitive sequences; Rate (%): the proportion of repetitive sequences in the total genome; the “0” represents a ratio that is less than 0.01.

### Protein-coding gene prediction and annotation

**Fig. 6:**
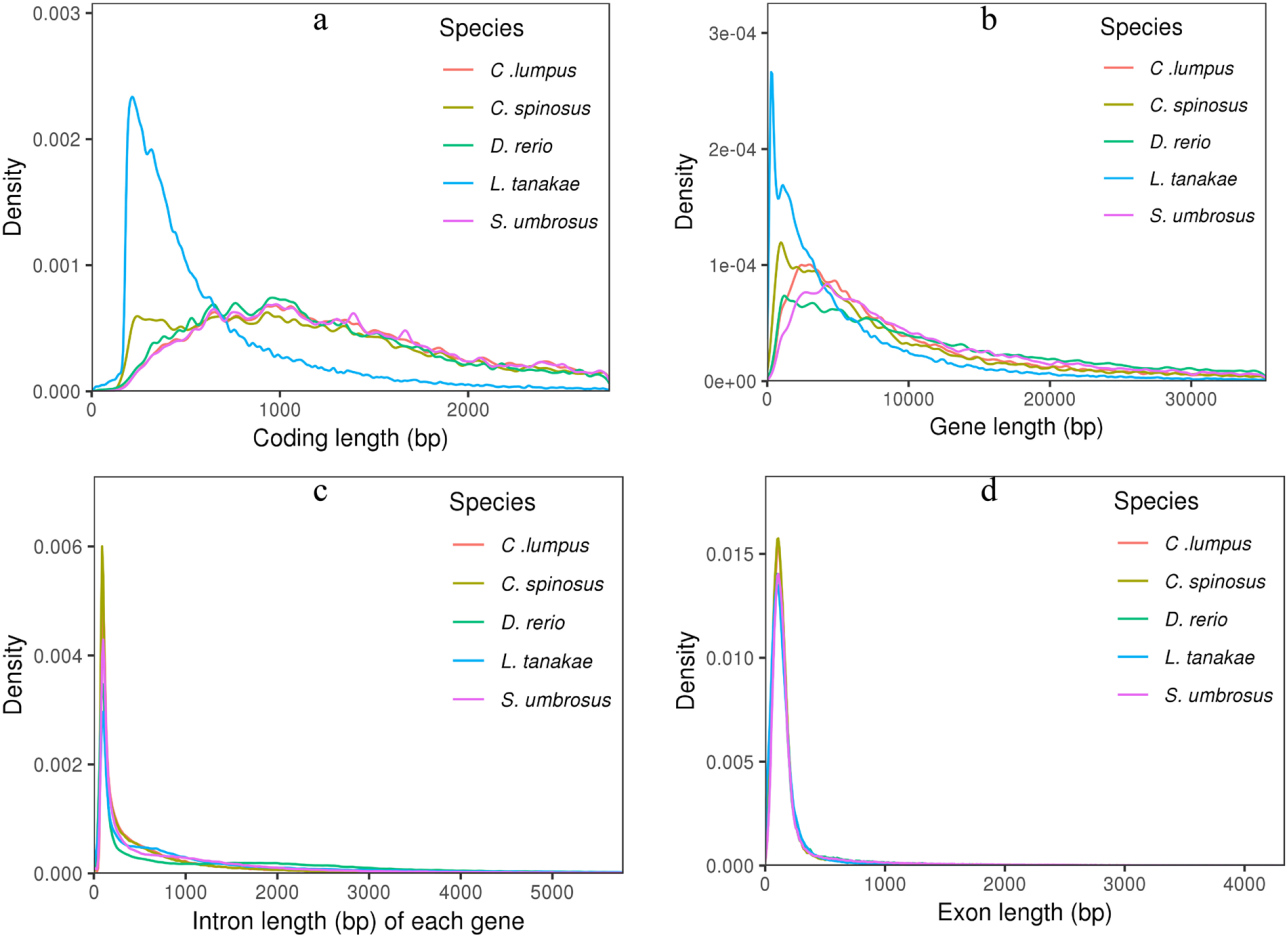
The composition of gene elements in the *C. spinosus* genome to other species. (**a**) Coding length distribution and comparison with other species. (**b**) Gene length distribution and comparison with other species. (**c**) Intron length distribution and comparison with other species. (**d**) Exon length distribution and comparison with other species.

We integrated three approaches, namely, de novo prediction, homology search, and transcript-based assembly, to annotate protein-coding genes in the genome. Firstly, the de novo gene models were predicted using two ab initio gene-prediction software tools, Augustus (version 2.4)^35^ and SNAP (2006-07-28)^36^. Secondly, GeMoMa (v 1.7)^37^ was performed for prediction based on homologous species. The protein sequences of *C. lumpus*, *D. rerio*, *L. tanakae*, and *S. umbrosus* were downloaded from GenBank and Ensembl database^38^. The protein sequences were aligned against the genome assembly using TBLASTN v2.2.2631^39^ (E-value ≤ 1e-5), and then matching proteins were aligned to the homologous genome sequences for accurate spliced alignments with GeneWise v2.4.132^40^. Thirdly, for the transcript-based prediction, RNA-sequencing data were mapped to the reference genome using Hisat (v 2.0.4)^41^ and assembled by Stringtie (v 1.2.3)^42^. GeneMarkS-T (v 5.1)^43^ was used to predict genes based on the assembled transcripts. The PASA (v 2.0.2)^44^ software was used to predict genes based on the unigenes (and full-length transcripts from the PacBio (ONT) sequencing) assembled by Trinity (v2.11)^45^. Finally, gene models from these different approaches were combined using the EVM software (v 1.1.1)^46^ and updated by PASA. The final gene models were annotated by searching the GenBank Non-Redundant (NR, 20200921), TrEMBL (202005), Pfam (33.1), SwissProt (202005), eukaryotic orthologous groups (KOG, 20110125), gene ontology (GO, 20200615) and Kyoto Encyclopedia of Genes and Genomes (KEGG, 20191220) databases. Overall, A total of 25,358 protein-coding genes were predicted by integrating the prediction of ab initio, homology-based, and RNA-seq strategies (Table 7), with an average gene length of 15,136 bp, exon length of 2,314 bp, coding sequence of 1,678 bp and intron length of 12,822 bp (Table 8). The statistics of gene models, including coding length, gene length, intron length, and exon length in *C. spinosus* were comparable to those for close-related species (Fig. 6). Ultimately, 24,072 genes (94.93% of the total) were successfully annotated GO, KEGG, KOG, Pfam, Swissprot, TrEMBL, EggNOG and NR database (Table 9).

**Table 7:** Gene annotation of *C. spinosus* genome via three methods.

Method	Gene set	Species	Number
Ab initio	Augustus	—	26,453
	SNAP	—	57,975
Homology-based	GeMoMa	*C. lumpus*	18,793
		*D. rerio*	9,703
		*L. tanakae*	25,565
		*S. umbrosus*	18,796
RNA-seq	GeneMarkS-T	—	20,356
	PASA	—	19,983
Integration	EVM	—	25,358

**Table 8:** The comparison of gene models annotated from the *C. spinous* genome with those from teleost fishes.

Species	*C. spinosus*	*D. rerio*	*S. umbrosus*	*L. tanakae*	*C. lumous*
Gene Num	25,358	32,147	23,703	68,268	21,142
Gene Len	383,812,559	975,484,643	540,860,487	459,402,160	326,235,140
Ave Gen Len	15135.76	30344.5	22818.23	6729.39	15430.67
Exon Len	58,670,195	86,529,582	76,804,477	52,714,163	61,275,739
Ave Exon Len	2313.68	2691.69	3240.29	772.17	2898.29
Exon Num	252,324	322,776	278,726	298,740	247,920
Ave Exon Num	9.95	10.04	11.76	4.38	9.95
CDS Len	42,547,416	54,528,638	43,390,472	45,858,516	38,274,768
Ave CDS Len	1677.87	1696.23	1830.59	671.74	1810.37
CDS Num	247,195	301,861	247,035	293,464	226,044
Ave CDS Num	9.75	9.39	10.42	4.3	9.75
Intron Len	325,142,364	888,955,061	464,056,010	406,687,997	264,959,401
Ave Intron Len	12822.08	27652.82	19577.94	5957.23	12532.37
Intron Num	226,966	290,397	254,368	230,472	226,655
Ave Intron Num	8.95	9.03	10.73	3.38	10.72

**Table 9:** Gene function annotation statistics of the assembled genome for *C. spinosus*.

Annotation database	Annotated number	Percentage (%)
GO	20,472	80.73
KEGG	20,481	80.77
KOG	15,704	61.93
Pfam	21,451	84.59
Swissprot	21,448	84.58
TrEMBL	23,607	93.09
EggNOG	20,368	80.32
NR	23,694	93.44
All Annotated	24,072	94.93

### Noncoding RNAs annotation and Pseudogene prediction

Non-coding RNAs are usually divided into several groups, including miRNA, rRNA, tRNA, snoRNA, and snRNA. The tRNAscan-SE (v 1.3.1)^47^ was used to predict tRNA with eukaryote parameters. Identification of the rRNA genes was conducted by Barrnap (v 0.9)^48^ based on Rfam (v 12.0) database^49^. miRNA genes were identified by searching miRBase (v 21) databases^50^. The snoRNA and snRNA genes were predicted using INFERNAL (v 1.1)^51^ based on the Rfam database. Finally, a total of 6,326 tRNAs, 962 rRNAs, and 947 miRNAs were predicted (Table 10).

**Table 10:** Noncoding RNA and pseudogene statistics of the assembled genome.

RNA classification	Number	Pseudogene	Value
miRNA	947	Number	82
rRNA	962	Total length	335,565
tRNA	6,326	Average length	4092.26

Pseudogenes typically exhibit sequences similar to functional genes but may lose their biological function due to genetic mutations, such as insertions and deletions. To identify such pseudogenes, we performed a comprehensive genome scan using the GenBlastA program (v 1.0.4)^52^ after excluding predicted functional genes. Subsequently, we employed GeneWise (v 2.4.1)^53^ to search for immature mutations and frameshift mutations to analyze the putative candidate genes. As a result, a total of 82 pseudogenes were identified, encompassing a combined length of 335,565 base pairs, with an average length of 4,092 base pairs (Table 10).

## Data Records

The sequencing dataset and genome assembly were deposited in public repositories. Illumina, PacBio, Hi-C and RNA-seq sequencing data used for Genome assembly have been deposited in the Genome Sequence Archive (GSA) at the National Genomics Data Center (NGDC)/China National Center for Bioinformation (CNCB) under accession number CRA009849^54^. This Whole Genome Shotgun project has been deposited at GenBank under the accession JARXKY000000000^55^. The version described in this paper is version JARXKY000000000.1^55^. Moreover, the genomic annotation results have been deposited in the *figshare* database^56^.

## Technical Validation

The assembly was evaluated using three criteria: the mapping of Illumina reads, core gene integrity, and BUSCO assessment. The reads from the short-insert library were re-mapped onto the assembly using BWA^21^. The assembly completeness was evaluated using Core Eukaryotic Genes Mapping Approach (CEGMA) software (version 2.5)^57^ and Benchmarking Universal Single-Copy Orthologs (BUSCO) software (version 2.0)^58^. The Illumina reads fully (98.94%) mapped to the assembled genome, including 97.23% of paired-end reads (Table 11). A total of 453 out of 458 conserved eukaryotic core genes from the CEGMA database were found in the assembled genome. Finally, 97.44% of the complete BUSCOs were included in the assembled genome (Table 12).

**Table 11:** Results of Illumina reads mapped to the *C. spinosus* assembly genome.

Type	Total reads	Mapped reads	Mapped (%)	Properly mapped reads	Properly mapped (%)
Length (bp)	708,955,931	701,453,038	98.94	685,018,428	97.23

**Table 12:** Results of the CEGMA and BUSCO assessment of *C. spinosus*.

Type		Number (percent)
CEGMA assessment	Number of 458 CEGMA present in the assembly	453 (98.91%)
	Number of 248 highly conserved CEGMA present	225 (90.73%)
BUSCO assessment	Complete and single-copy BUSCOs (S)	908 (92.84%)
	Complete and duplicated BUSCOs (D)	45 (4.60%)
	Fragmented BUSCOs (F)	5 (0.51%)
	Missing BUSCOs (M)	20 (2.04%)
	Total Lineage BUSCOs	978

## Data Availability

All commands and pipelines used in data processing were executed according to the manual and protocols of the corresponding bioinformatics software.

## References

[1] Ni, Y. & Wu, H. *Fishes of Jiangsu Province* (China Agriculture Press, 2006).

[2] Zhang Y (2018). The distribution and biological characteristics of *Chelidonichthy Kumu* in the North East China Sea. J. Zhejiang Univ..

[3] FAO. *The State of World Fisheries and Aquaculture 2018 - Meeting the sustainable development goals* (Food and Agriculture Organization of the United Nations, 2018).

[4] Liang C, Xian WW, Liu SD, Pauly D (2020). Assessments of 14 exploited fish and invertebrate stocks in chinese waters using the LBB method. Front. Mar. Sci..

[5] Wang RF (2019). Study on spatial heterogeneity in feeding habits of *Chelidonichthys spinosus* in Haizhou Bay during autumn. Acta. Ecologica. Sinica..

[6] Zhang ZX, Mammola S, Xian WW, Zhang H (2020). Modelling the potential impacts of climate change on the distribution of ichthyoplankton in the Yangtze Estuary, China. Divers. Distrib..

[7] National Animal Collection Resource Center. *Zoology and Local Chronicles of China in 2017.* http://museum.ioz.ac.cn/topic_detail.aspx?id=68958 (2020).

[8] Zhuang, P. *Fishes of the Yangtze Estuary*. (China Agriculture Press, 2006).

[9] National Center for Biotechnology Information. *Genome.* https://www.ncbi.nlm.nih.gov/genome/?term=fish (2023).

[10] Jones FC (2012). The genomic basis of adaptive evolution in three spine sticklebacks. Nature.

[11] Lin Q (2016). The seahorse genome and the evolution of its specialized morphology. Nature.

[12] Shao CW (2018). Chromosome-level genome assembly of the spotted sea bass, *Lateolabrax maculates*. Gigascience.

[13] Chen BH (2019). The sequencing and de novo assembly of the *Larimichthys crocea* genome using PacBio and Hi-C technologies. Sci. Data.

[14] Liu ZY (2022). Chromosomal fusions facilitate adaptation to divergent environments in threespine stickleback. Mol. Biol. Evol..

[15] Korlach J (2010). Real-time DNA sequencing from single polymerase molecules. Methods Enzymol..

[16] Rao SSP (2015). A 3D map of the human genome at kilobase resolution reveals principles of chromatin looping (vol 159, pg 1665, 2014). Cell.

[17] Marcais G, Kingsford C (2011). A fast, lock-free approach for efficient parallel counting of occurrences of k-mers. Bioinformatics.

[18] Ruan J, Li H (2020). Fast and accurate long-read assembly with wtdbg2. Nat. Methods.

[19] Chin CS (2013). Nonhybrid, finished microbial genome assemblies from long-read SMRT sequencing data. Nat. Methods.

[20] Walker BJ (2014). Pilon: An Integrated Tool for Comprehensive Microbial Variant Detection and Genome Assembly Improvement. Plos One.

[21] Li H, Durbin R (2010). Fast and accurate long-read alignment with Burrows-Wheeler transform. Bioinformatics.

[22] Servant N (2015). HiC-Pro: an optimized and flexible pipeline for Hi-C data processing. Genome Biol..

[23] Burton JN (2013). Chromosome-scale scaffolding of de novo genome assemblies based on chromatin interactions. Nat. Biotechnol..

[24] Flynn JM (2020). RepeatModeler2 for automated genomic discovery of transposable element families. PNAS..

[25] Bao ZR, Eddy SR (2002). Automated de novo identification of repeat sequence families in sequenced genomes. Genome Res..

[26] Price AL, Jones NC, Pevzner PA (2005). De novo identification of repeat families in large genomes. Bioinformatics.

[27] Ellinghaus D, Kurtz S, Willhoeft U (2008). LTRharvest, an efficient and flexible software for de novo detection of LTR retrotransposons. Bmc Bioinformatics.

[28] Xu Z, Wang H (2007). LTR_FINDER: an efficient tool for the prediction of full-length LTR retrotransposons. Nucleic Acids Res..

[29] Ou SJ, Jiang N (2018). LTR_retriever: A highly accurate and sensitive program for identification of long terminal repeat retrotransposons. Plant Physiol..

[30] Jurka J (2005). Repbase update, a database of eukaryotic repetitive elements. Cytogenet Genome Res..

[31] Neumann P, Novak P, Hostakova N, Macas J (2019). Systematic survey of plant LTR-retrotransposons elucidates phylogenetic relationships of their polyprotein domains and provides a reference for element classification. Mobile DNA.

[32] Wheeler TJ (2013). Dfam: a database of repetitive DNA based on profile hidden Markov models. Nucleic Acids Res..

[33] Tarailo-Graovac M, Chen N (2009). Using RepeatMasker to identify repetitive elements in genomic sequences. Curr. Protoc. Bioinform..

[34] Beier S, Thiel T, Munch T, Scholz U, Mascher M (2017). MISA-web: a web server for microsatellite prediction. Bioinformatics.

[35] Stanke M, Diekhans M, Baertsch R, Haussler D (2008). Using native and syntenically mapped cDNA alignments to improve de novo gene finding. Bioinformatics.

[36] Korf I (2004). Gene finding in novel genomes. Bmc Bioinformatics.

[37] Keilwagen J (2016). Using intron position conservation for homology-based gene prediction. Nucleic Acids Res..

[38] Cunningham F (2019). Ensembl 2019. Nucleic Acids Res..

[39] Gertz EM, Yu YK, Agarwala R, Schaffer AA, Altschul SF (2006). Composition-based statistics and translated nucleotide searches: Improving the TBLASTN module of BLAST. Bmc Biol..

[40] Doerks T, Copley RR, Schultz J, Ponting CP, Bork P (2002). Systematic identification of novel protein domain families associated with nuclear functions. Genome Res..

[41] Kim D, Landmead B, Salzberg SL (2015). HISAT: a fast spliced aligner with low memory requirements. Nat. Methods.

[42] Pertea M (2015). StringTie enables improved reconstruction of a transcriptome from RNA-seq reads. Nat. Biotechnol..

[43] Tang SYY, Lomsadze A, Borodovsky M (2015). Identification of protein coding regions in RNA transcripts. Nucleic Acids Res..

[44] Haas BJ (2003). Improving the Arabidopsis genome annotation using maximal transcript alignment assemblies. Nucleic Acids Res..

[45] Grabherr MG (2011). Full-length transcriptome assembly from RNA-Seq data without a reference genome. Nat. Biotechnol..

[46] Haas BJ (2008). Automated eukaryotic gene structure annotation using EVidenceModeler and the program to assemble spliced alignments. Genome Biol..

[47] Lowe TM, Eddy SR (1997). tRNAscan-SE: A program for improved detection of transfer RNA genes in genomic sequence. Nucleic Acids Res..

[48] Loman, T. *A Novel Method for Predicting Ribosomal RNA Genes in Prokaryotic Genomes.* http://lup.lub.lu.se/student-papers/record/8914064 (2017).

[49] Griffiths-Jones S (2005). Rfam: annotating non-coding RNAs in complete genomes. Nucleic Acids Res..

[50] Griffiths-Jones S, Grocock RJ, van Dongen S, Bateman A, Enright AJ (2006). miRBase: microRNA sequences, targets and gene nomenclature. Nucleic Acids Res..

[51] Nawrocki EP, Eddy SR (2013). Infernal 1.1: 100-fold faster RNA homology searches. Bioinformatics.

[52] She R, Chu JSC, Wang K, Pei J, Chen NS (2009). GenBlastA: Enabling BLAST to identify homologous gene sequences. Genome Res..

[53] Birney E, Clamp M, Durbin R (2004). GeneWise and Genomewise. Genome Res..

[54] *NGDC/CNCB Genome Sequence Archive* https://ngdc.cncb.ac.cn/gsa/browse/CRA009849 (2023).

[55] Wang Y, Zhang H, Xian W, Iwasaki W (2023). GenBank.

[56] Wang Y (2023). figshare.

[57] Parra G, Bradnam K, Korf I (2007). CEGMA: a pipeline to accurately annotate core genes in eukaryotic genomes. Bioinformatics.

[58] Simao FA, Waterhouse RM, Ioannidis P, Kriventseva EV, Zdobnov EM (2015). BUSCO: assessing genome assembly and annotation completeness with single-copy orthologs. Bioinformatics.

